# miR‐222 attenuates cisplatin‐induced cell death by targeting the PPP2R2A/Akt/mTOR Axis in bladder cancer cells

**DOI:** 10.1111/jcmm.12760

**Published:** 2016-01-22

**Authors:** Li‐Ping Zeng, Zheng‐Mao Hu, Kai Li, Kun Xia

**Affiliations:** ^1^ The State Key Laboratory of Medical GeneticsCentral South UniversityChangshaHunanChina; ^2^ The Clinical Laboratory of No.261 Hospital of the People's Liberation ArmyBeijingChina

**Keywords:** microRNA‐222, bladder cancer, cisplatin

## Abstract

Increased miR‐222 levels are associated with a poor prognosis in patients with bladder cancer. However, the role of miR‐222 remains unclear. In the present study, we found that miR‐222 enhanced the proliferation of both the T24 and the 5637 bladder cancer cell lines. Overexpression of miR‐222 attenuated cisplatin‐induced cell death in bladder cancer cells. miR‐222 activated the Akt/mTOR pathway and inhibited cisplatin‐induced autophagy in bladder cancer cells by directly targeting protein phosphatase 2A subunit B (PPP2R2A). Blocking the activation of Akt with LY294002 or mTOR with rapamycin significantly prevented miR‐222‐induced proliferation and restored the sensitivity of bladder cancer cells to cisplatin. These findings demonstrate that miR‐222 modulates the PPP2R2A/Akt/mTOR axis and thus plays a critical role in regulating proliferation and chemotherapeutic drug resistance. Therefore, miR‐222 may be a novel therapeutic target for bladder cancer.

## Introduction

MicroRNAs have emerged as important regulators of proliferation, migration, invasion and apoptosis in cancer cells. Previously, microRNA‐222 (miR‐222) has been reported to be up‐regulated in various human cancers, including prostate cancer 1, nonsmall cell lung cancer 2, breast cancer 3, hepatocellular cancer 4 and bladder cancer 5. Through targeting tumour suppressors, such as p27 6, TIMP3 7 and protein phosphatase 2A subunit B (PPP2R2A) 4, miR‐222 promotes the proliferation, migration and invasion of cancer cells. Importantly, miR‐222 participates in the regulation of sensitivity to both hormone‐based and conventional chemotherapy 8, indicating that miR‐222 may modulate the response to cancer therapy. The up‐regulation of miR‐222 in bladder cancer is associated with a poor prognosis 5. Therefore, we hypothesized that miR‐222 might promote cancer growth, attenuate the effects of chemotherapy and thus exacerbate the poor prognosis of bladder cancer.

For patients with advanced bladder cancer, the first‐line treatment is cisplatin (CDDP)‐based chemotherapy. However, most of these patients lose the response to CDDP and eventually die of the disease 9. Therefore, the identification of novel regulators of CDDP resistance in bladder cancer cells is critical. In the present study, we found that miR‐222 promoted the proliferation of bladder cancer cells and attenuated CDDP‐induced cell death by regulating the PPP2R2A/Akt/mTOR axis, which indicated that miR‐222 might be a novel therapeutic target for bladder cancer.

## Materials and methods

### Reagents and cell culture

CDDP and rapamycin were purchased from Sigma‐Aldrich (St. Louis, MO, USA). A 1 mg/ml stock solution of CDDP was made in PBS, stored at −20°C and diluted in fresh medium to various concentrations prior to use. Rapamycin was dissolved in dimethyl sulphoxide (DMSO) in a 1 μM stock solution and stored at −20°C. Foetal bovine serum (FBS), antibiotic mixtures, trypsin and PBS were purchased from Life Technologies (Carlsbad, CA, USA). Rabbit anti‐p‐Akt, Akt, p‐p70S6K, p70S6K, p62, cleaved caspase 3, GAPDH and mouse anti‐PPP2R2A were purchased from Cell Signaling Technology (Beverly, MA, USA). Rabbit anti‐LC3B was purchased from Sigma‐Aldrich. LY294002 was purchased from Cell Signaling Technology. T24 and 5637 cell lines were cultured in RPMI‐1640 medium (Life Technologies) containing 10% FBS, 100 units/ml penicillin and 0.1 mg/ml streptomycin. Cells were maintained in an atmosphere of 5% CO_2_ at 37°C.

### Plasmid construction

Total RNA from HEK 293 cells was synthesized into cDNA for amplification. The coding sequence of PPP2R2A was amplified by using the following primers: forward: 5′‐CCAAGCTTATGGCAGGAGCTGGAGG‐3′ and reverse: 5′‐CCGGAATTCCTAATTCACTTTGTCTTGAAATATA‐3′. The PCR products and the pcDNA3.1 vector were purified and digested with *Hind*III and *Eco*RI (TaKaRa, Dalian, China). Then, the fragment was cloned into the pcDNA3.1 vector to construct the pcDNA3.1‐PPP2R2A plasmid. The plasmid was amplified, extracted and purified for transfection.

### Cell treatments

Cells were starved in FBS‐free RPMI‐1640 medium for 24 hrs and then transiently transfected with the miR‐222 mimic or antagomir (GenePharma, Shanghai, China) at a final concentration of 100 nM with Lipofectamine 2000 (Life Technologies) to overexpress or knock down miR‐222, respectively, according to the manufacturer's protocol. Then, the supernatant was replaced with complete RPMI‐1640 medium and the cells were incubated for 48 hrs. For CDDP treatment, the cells were incubated with various concentrations of CDDP for 24 hrs.

### Cell proliferation assay

Cell viability in each group was measured by using CCK‐8 (Dojindo, Kumamoto, Japan). The cells were seeded into 96‐well plates at a density of 1 × 10^4^ cells/well, cultured overnight and then treated as described above. The Cell Counting Kit‐8 (CCK‐8) reagent was added to each well according to the manufacturer's instructions. Then, the plates were incubated at 37°C for 2 hrs, and the optical densities were read at 450 nm.

### Cell death analysis

To measure cell death, a FITC Annexin‐V apoptosis detection kit (BD Biosciences, Franklin Lakes, NJ, USA) was used according to the manufacturer's instructions. Briefly, the cells were harvested and washed with using ice‐cold PBS, stained with Annexin V/propidium iodide (PI) at room temperature in the dark and then detected with a flow cytometer (FACSCalibur; BD Biosciences). All flow cytometry data were analysed with the FlowJO software program.

### Western blot analysis

Whole‐cell lysates were prepared in RIPA lysis buffer (50 mM Tris‐HCl, pH 7.4, 150 mM NaCl, 1 mM ethylenediaminetetraacetic acid, 1% NP‐40, and 0.1% sodium dodecyl sulphate), quantified and loaded onto SDS‐PAGE gels. After electrophoresis, the proteins were transferred to a nitrocellulose membrane and blocked with 5% nonfat milk in Tris‐buffered saline (TBS)/Tween (0.05% Tween‐20 in TBS; TBST). Then the membrane was incubated with the corresponding primary antibodies at 4°C overnight. The membrane was washed in TBST buffer and incubated with an horseradish peroxidase (HRP)‐conjugated secondary antibody for 1 hr at room temperature. After 5 washes with TBST, the immunoblots were detected by using an HRP substrate (Millipore, Bedford, MA, USA) and visualized using a G:BOX Chemi Gel Documentation System (Syngene, Cambridge, UK).

### Dual luciferase assay

The 3′UTR of human PPP2R2A‐containing miR‐222 target sites was amplified and cloned into the pMIR‐REPORT vector (Life Technologies) to generate the wild‐type pMIR‐PPP2R2A‐3′UTR reporter. A mutant fragment with the miR‐222 target site was synthesized and cloned into the same vector to generate a mutated pMIR‐ PPP2R2A‐3′UTR plasmid. Both the wild‐type and mutant constructs were verified by sequencing. The constructs, microRNA mimic and pRL‐TK were cotransfected with Lipofectamine 2000 into T24 cells. The cells were lysed and were collected 48 hrs after transfection. The luciferase activity was measured by using the Dual Luciferase Reporter Assay Kit (Promega, Madison, WI, USA) with a luminometer (Thermo, Waltham, MA, USA).

### Confocal microscopy assay

T24 cells were transfected with the EGFP‐LC3 plasmid for 48 hrs, and EGFP‐LC3 puncta were visualized and counted by using confocal microscopy (FV1000; Olympus, Tokyo, Japan). The number of LC3 puncta per cell was calculated.

### Statistical analysis

Data were presented as the mean ± S.D. of at least three independent experiments. A two‐tailed Student's *t*‐test or an anova least significant difference test was performed using SPSS 19.0 software (SPSS Inc., Chicago, IL, USA) to determine the statistical significance of the results. A *P* < 0.05 was considered to be statistically significant.

## Results

### miR‐222 promotes the proliferation of bladder cancer cells

**Figure 1:**
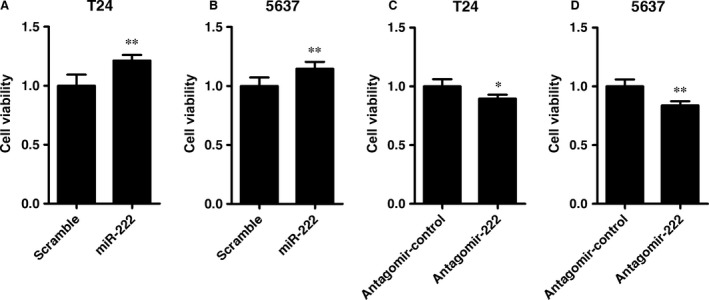
miR‐222 promotes the proliferation of bladder cancer cells. (**A**–**D**) T24 (**A** and **C**) and 5637 cells (**B** and **D**) were transfected with the miR‐222 mimics or antagomir. Cell viability was determined by using the Cell Counting Kit‐8 assay 48 hrs after transfection. Data are shown as the mean ± S.D. (*n* = 5 per group). *P < 0.05 and **P < 0.01 *versus* the scrambled RNA (**A** and **B**) or Antagomir‐control group (**C** and **D**).

To determine whether miR‐222 promoted the proliferation of bladder cancer cells, we transfected a miR‐222 mimic or antagomir into T24 and 5637 cells for 48 hrs. The miR‐222 levles in T24 and 5637 cells transfected with the miR‐222 mimic were increased to 20.1‐ and 22.8‐fold compared with their corresponding control cells. In contrast, the miR‐222 levels dropped to 40.7% and 49.6% compared with the control cells after transfected with the miR‐222 antagomir. Cell viability was detected by using the CCK‐8 assay. We observed that the viability was significantly increased to 1.12‐ and 1.45‐fold in T24 and 5637 cells transfected with the miR‐222 mimic, respectively, compared with that in the control cells (Fig. 1A and B). In contrast, the viability of T24 and 5637 cells transfected with the miR‐222 antagomir decreased to 89.6% and 83.7%, respectively, compared with that in control cells (Fig. 1C and D). These results demonstrated that miR‐222 promoted the proliferation of bladder cancer cells.

### miR‐222 induces resistance of bladder cancer cells to cisplatin

**Figure 2:**
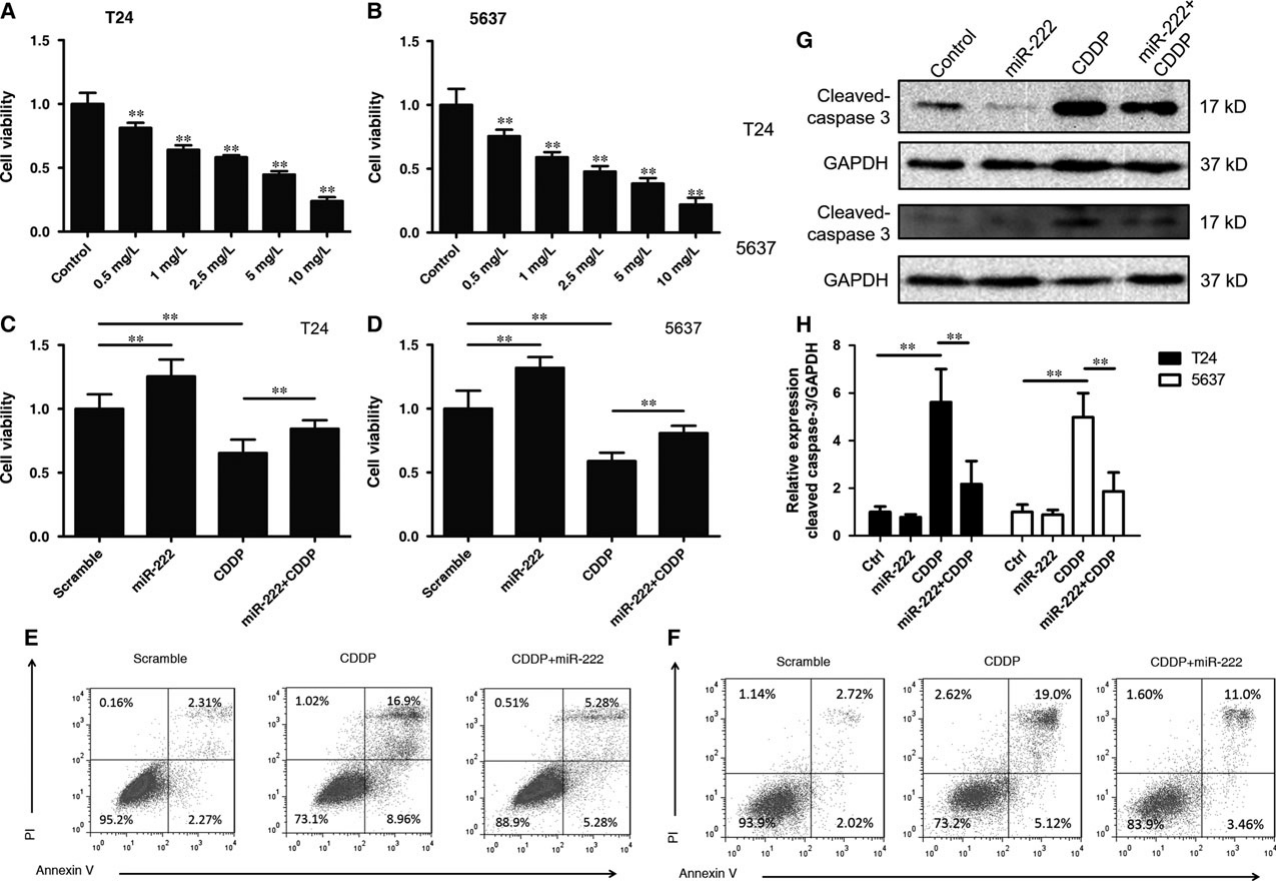
miR‐222 inhibits cisplatin‐induced cell death in bladder cancer cells. (**A** and **B**) T24 (**A**) or 5637 (**B**) cells were treated with cisplatin (CDDP) for 24 hrs, and cell viability was detected using the Cell Counting Kit‐8 (CCK‐8) assay. (**C**–**F**) T24 (**C** and **E**) or 5637 (**D** and **F**) cells were transfected with the miR‐222 mimics or scrambled RNA. After 24 hrs after transfection, the cells were treated with CDDP (2.5 mg/l) for another 24 hrs, and the CCK‐8 assay (**C** and **D**) or flow cytometry assay (**E** and **F**) was performed. (**G** and **H**) The cells were treated as described above (**C**–**F**), and Western blotting was performed to detect the cleaved form of caspase‐3. **P < 0.01 *versus* the control group (**A** and **B**) or the indicated groups (**C**,** D** and **H**).

Because miR‐222 mediates chemotherapy resistance in many cancers 8, we measured whether miR‐222 also mediated chemotherapy resistance in bladder cancer cells. CDDP is a commonly used chemotherapy drug for advanced bladder cancer. We incubated T24 and 5637 cells with a range of CDDP concentrations for 24 hrs. We observed that the viability of both the T24 and 5637 cell lines was inhibited by CDDP in a concentration‐dependent manner (Fig. 2A and B). The IC50 value of CDDP at 24 hrs was 2.95 mg/l in the T24 cells and 2.08 mg/l in the 5637 cells. Because both of these cell lines showed significant sensitivity towards 2.5 mg/l CDDP, we selected this concentration for the following analyses. We transiently transfected miR‐222 mimics into the two cell lines cotreated with CDDP (2.5 mg/l). We observed that overexpression of miR‐222 significantly inhibited CDDP‐induced cell death in both cell lines (Fig. 2C and D).

Flow cytometry was performed to detect whether CDDP could induce cell death *in vitro*. T24 and 5637 cells were transfected with the miR‐222 mimic for 24 hrs. Then, the cells were incubated with CDDP (2.5 mg/l) for another 24 hrs. We observed that miR‐222 overexpression partially attenuated CDDP‐induced cell death (Fig. 2E and F). The cleaved form of caspase‐3 is a biomarker of apoptosis. We observed that miR‐222 overexpression suppressed the CDDP‐induced cleavage of caspase‐3 (Fig. 2G and H). These results indicated that miR‐222 attenuated the anticancer effects of CDDP in bladder cancer cells.

### miR‐222 targets protein phosphatase 2A subunit B in bladder cancer cells

**Figure 3:**
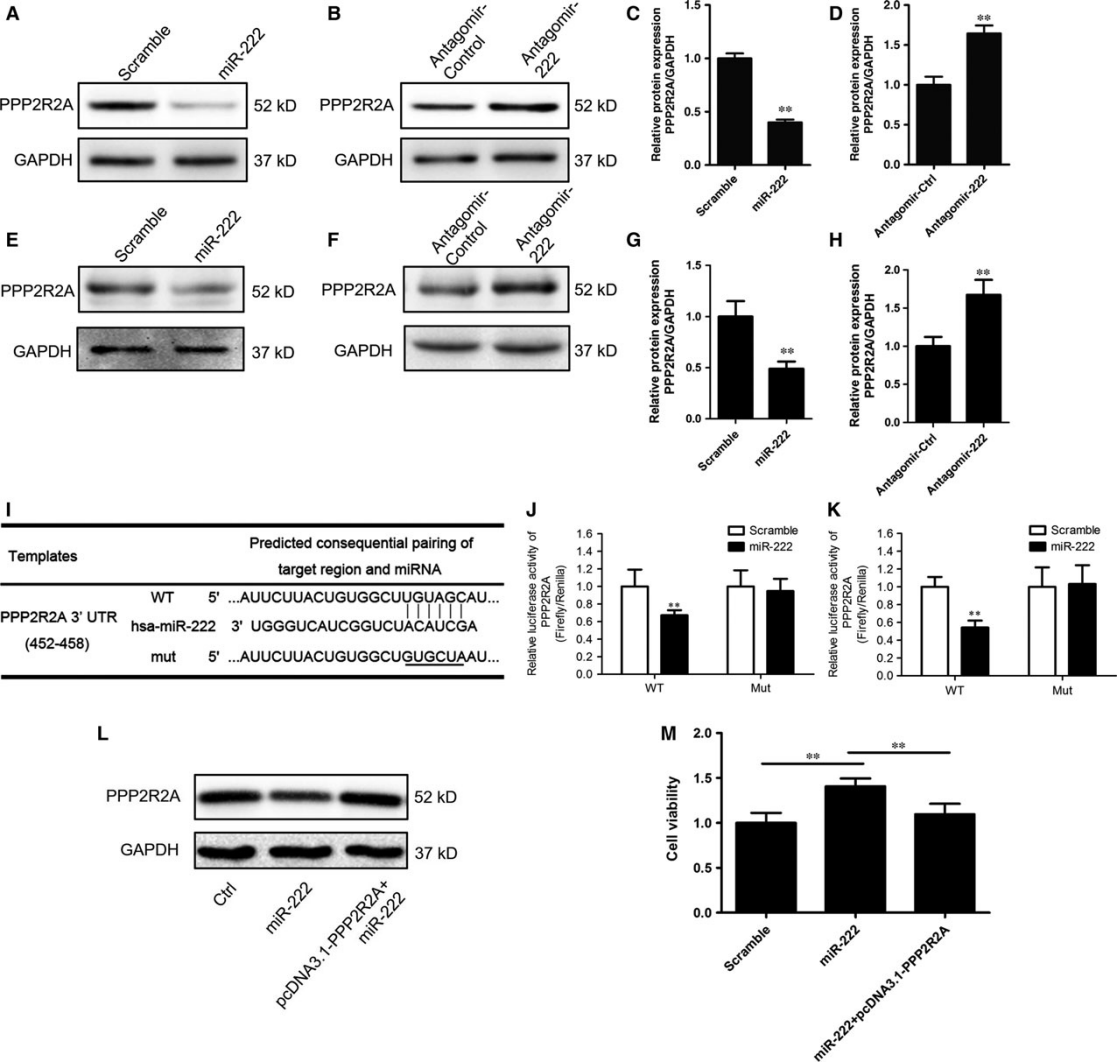
miR‐222 directly targets protein phosphatase 2A subunit B in bladder cancer cells. (**A**–**H**) The expression of protein phosphatase 2A subunit B (PPP2R2A) in T24 (**A**–**D**) and 5637 (**E**–**H**) cells transfected with the miR‐222 mimics (**A** and **E**) or antagomir (**B** and **F**). The relative expression of PPP2R2A was normalized to GAPDH (**C**,** D**,** G** and **H**). (**I**) PPP2R2A is predicted to be a potential target gene of miR‐222 by Targetscan. The miR‐222 binding sites in the wild‐type (WT) and mutated (mut) PPP2R2A 3′UTR sequences are shown. (**J** and **K**) The WT and Mut 3′UTR of PPP2R2A were cloned into pMIR‐REPORT vectors and cotransfected into T24 (**J**) and 5637 (**K**) cells with pRL‐TK and the miR‐222 mimics or scrambled RNA. The relative luciferase activity was measured and normalized to that of Renilla luciferase. (**L** and **M**) T24 cells were transfected as indicated and CCK‐8 assays were performed (**M**). The expression of PPP2R2A in each group is shown (**L**). Values are expressed as the means ± S.D.; **P < 0.01 *versus* the control group (**C**,** D**,** G** and **H**) or between the indicated groups (**M**).

PPP2R2A is a known target of miR‐222 in hepatocellular cancer cells 4. To explore whether miR‐222 also regulated PPP2R2A expression in bladder cancer cells, Western blotting was performed to detect the PPP2R2A levels in T24 and 5637 cells transfected with the miR‐222 mimic or antagomir. We observed that miR‐222 overexpression significantly down‐regulated PPP2R2A expression, whereas knock‐down of endogenous miR‐222 up‐regulated the PPP2R2A level (Fig. 3A–H). Data from the dual luciferase assay showed that miR‐222 decreased the luciferase activity of wild‐type pMIR‐PPP2R2A‐3′UTR but not that of the mutated pMIR‐PPP2R2A‐3′UTR (Fig. 3I–K).

To explore the role of PPP2R2A in the miR‐222‐induced proliferation of bladder cancer cells, we constructed the pcDNA3.1‐PPP2R2A plasmid to rescue the decreased level of PPP2R2A induced by miR‐222 overexpression. This plasmid compensated for the intracellular levels of PPP2R2A (Fig. 3L). By using the CCK‐8 assays, we observed that PPP2R2A overexpression restored miR‐222‐induced proliferation in T24 cells (Fig. 3M). Together, these results indicated that PPP2R2A was a direct target of miR‐222 in bladder cancer cells in a manner associated with miR‐222‐induced proliferation.

### miR‐222 inhibited the sensitivity to cisplatin by regulating the PPP2R2A/Akt/mTOR signalling pathway in bladder cancer cells

**Figure 4:**
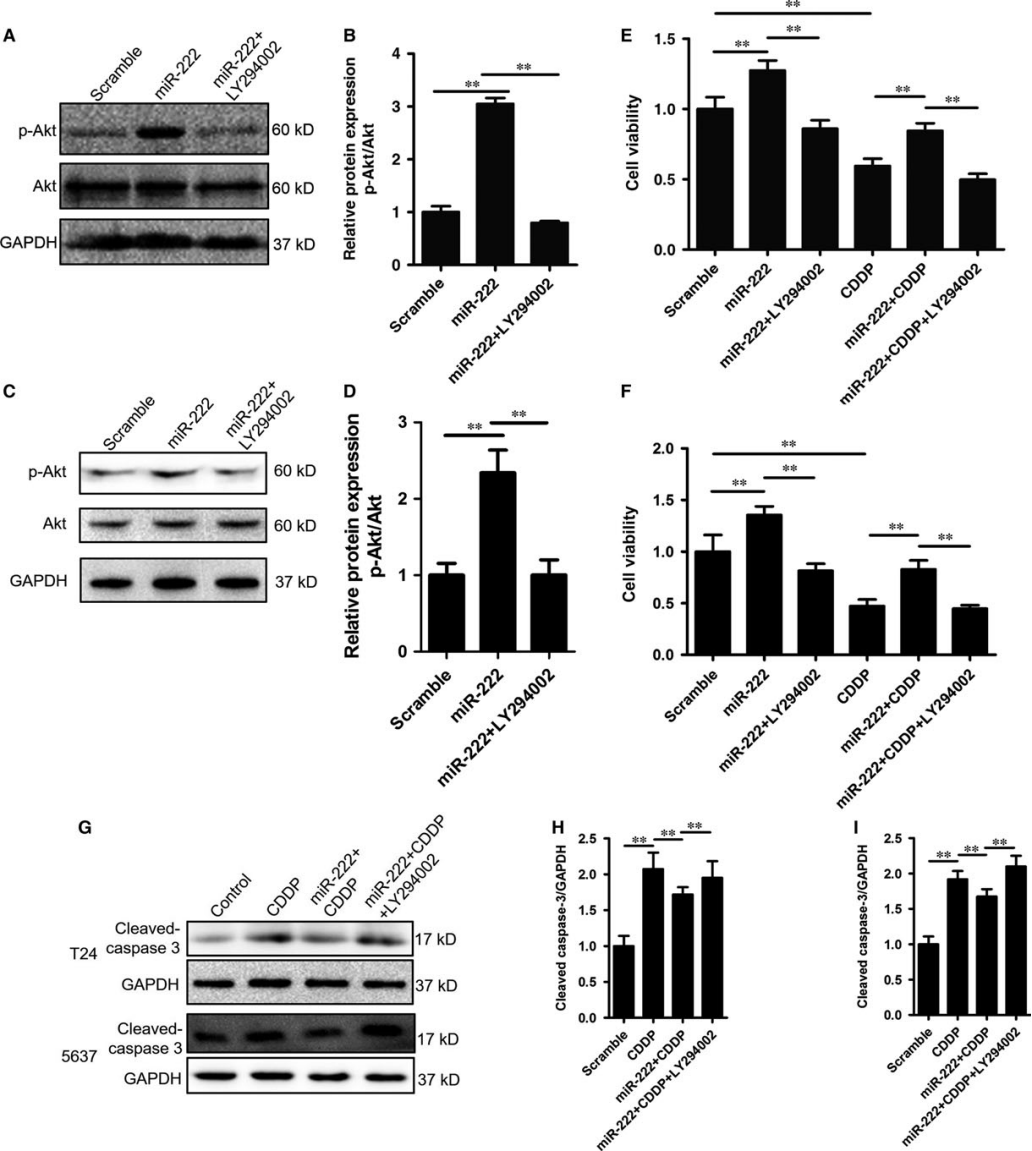
miR‐222 blocks cisplatin‐induced cell death through activation of the Akt pathway. (**A**–**D**) T24 (**A** and **B**) and 5637 (**C** and **D**) cells were treated as indicated and Western blotting was performed to detect the phosphorylation status of Akt (**A** and **C**) and the p‐Akt/Akt ratio (**B** and **D**). (**E** and **F**) T24 (**E**) and 5637 (**F**) cells were treated with LY294002 (20 μM) and/or CDDP (2.5 mg/l) as indicated, and cell viability was measured with Cell Counting Kit‐8 assay. (**G**–**I**) T24 (**G** and **H**) and 5637 (**G** and **I**) cells were treated as indicated, and the cleavage of caspase‐3 was detected by western blotting. Data are expressed as the mean ± S.D. of at least three independent experiments. **P < 0.01, between the indicated groups.

PPP2R2A is an important regulatory subunit of phosphatase 2A (PP2A) that catalyses the dephosphorylation of various kinases 10, 11, 12, 13, 14, 15. In hepatocellular carcinoma cells, miR‐222 targets PPP2R2A and thereby inhibits the activation of Akt 4. We evaluated the phosphorylation status of Akt in T24 and 5637 cells upon miR‐222 overexpression. We observed that the p‐Akt/Akt ratio in T24 and 5637 cells was indeed increased 24 hrs after transfection (Fig. 4A–D). To analyse whether the PPP2R2A/Akt axis played an important role in miR‐222‐mediated proliferation of bladder cancer cells, we used the specific PI3K inhibitor LY294002 to suppress Akt phosphorylation. We observed that LY294002 (20 μM) was sufficient to antagonize miR‐222‐induced Akt activation (Fig. 4A–D). Then, we transfected the miR‐222 mimic into T24 and 5637 cells for 24 hrs. The cells were cotreated with CDDP (2.5 mg/l) in the presence or absence of LY294002 (20 μM) for another 24 hrs. We observed that LY294002 was sufficient to block miR‐222‐induced proliferation (Fig. 4E and F). Furthermore, cotreatment with CDDP plus LY294002 resulted in lower viability compared with that upon treatment with CDDP alone in cells transfected with miR‐222 (Fig. 4E and F). Similarly, the cleaved form of caspase‐3 was increased in the presence of LY294002 (Fig. 4G–I).

**Figure 5:**
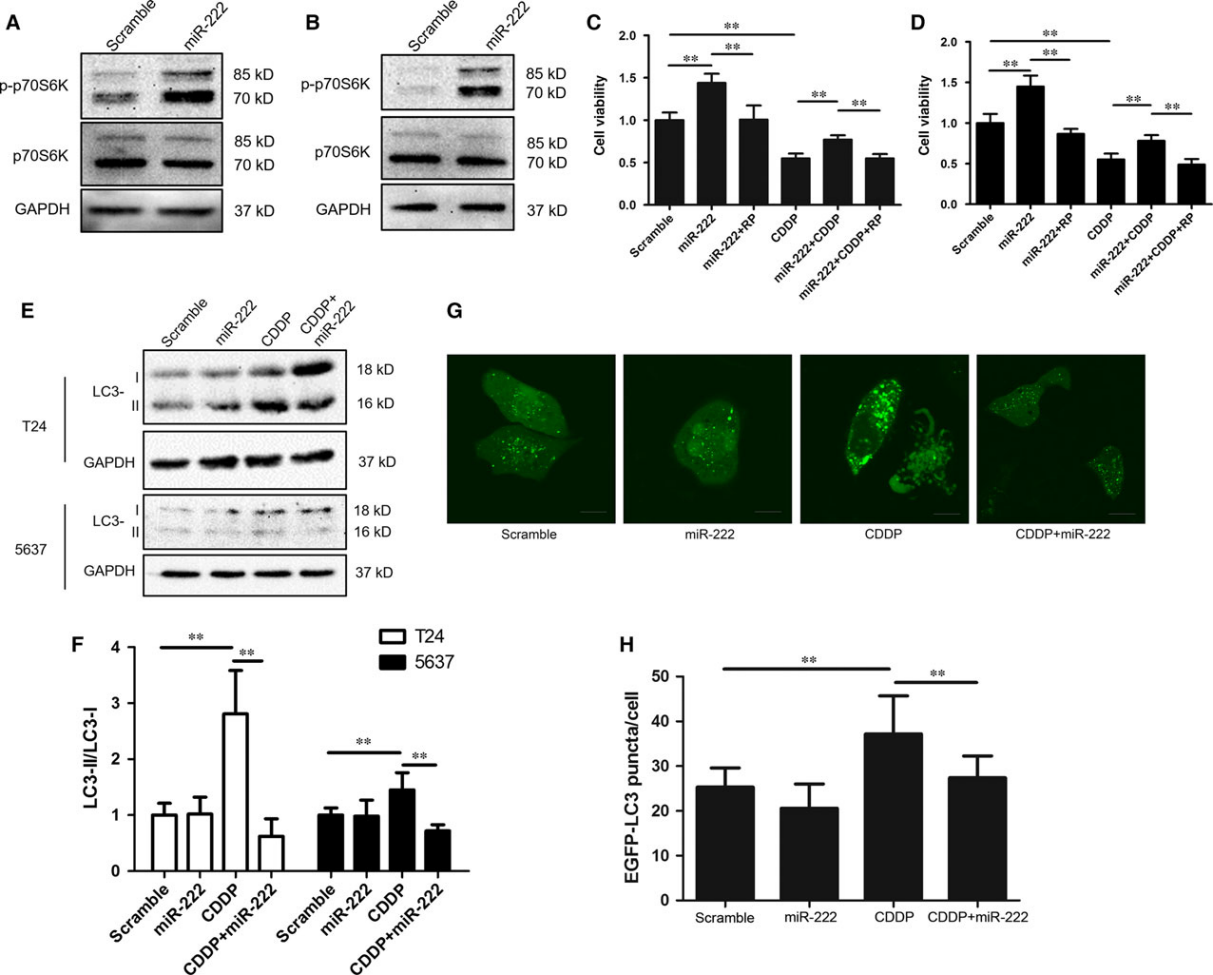
miR‐222 activates the mTOR pathway and inhibits autophagy in bladder cancer cells. (**A** and **B**) T24 (**A**) and 5637 (**B**) cells were transfected with the miR‐222 mimic and the phosphorylation of p70S6K 24 hrs after transfection was detected by using Western blotting. (**C** and **D**) T24 (**C**) and 5637 (**D**) cells were transfected as described above for 24 hrs. Rapamycin (RP, 20 nM) and/or CDDP (2.5 mg/l) were added as indicated, and the cells were incubated for 24 hrs. Cell viability were detected by using the Cell Counting Kit‐8 assay. (**E** and **F**) T24 cells were treated as described in (**A** and **B**), and LC3 lipidation was determined by Western blotting. (**G** and **H**) T24 cells were transfected with the EGFP‐LC3 plasmid and treated as indicated. The number of EGFP‐LC3 puncta was counted by using confocal microscopy. Data are expressed as the mean ± S.D. of at least three independent experiments. **P < 0.01, *versus* the indicated groups.

The Akt pathway induces mTOR activation, which is a known factor related with cell growth, metabolism, apoptosis and autophagy. To explore whether mTOR was activated, T24 cells were transfected with the miR‐222 mimic for 24 hrs, and the phosphorylation status of p70S6K (T389) was then detected. We observed a significant increase in the p70S6K phosphorylation levels in miR‐222‐transfected T24 and 5637 cells, indicating that miR‐222 could activate the mTOR pathway in bladder cancer cells (Fig. 5A and B). To analyse whether the activation of mTOR was required for miR‐222‐mediated CDDP resistance, we used the specific mTOR inhibitor rapamycin to block mTOR activity. We observed that rapamycin (20 nM) significantly antagonized the miR‐222‐induced proliferation and CDDP resistance (Fig. 5C and D).

mTOR is a known negative regulator of autophagy, whereas CDDP induces antitumorigenic autophagy in cancer cells 16. Therefore, we postulated that miR‐222 might inhibit autophagy in bladder cancer cells. We measured the expression of LC3 in bladder cancer cells and observed that miR‐222 overexpression did not significantly alter the LC3 lipidation compared with the baseline of T24 and 5637 cells (Fig. 5E and F). Similarly, the overexpression of miR‐222 in T24 cells did not change the number of LC3 puncta (Fig. 5G and H). However, miR‐222 overexpression dramatically antagonized CDDP‐induced autophagy in both T24 and 5637 cells, as demonstrated by the decreased LC3‐II levels (Fig. 5E and F) and number of LC3 puncta (Fig. 5G and H). These results indicated that miR‐222 might block CDDP‐induced autophagy in bladder cancer cells.

Together, these data demonstrate that the PPP2R2A/Akt/mTOR axis is required for miR‐222‐mediated proliferation and CDDP‐induced cell death in bladder cancer cells.

## Discussion

MicroRNAs play crucial roles in regulating proliferation in bladder cancer 5, 17, 18, 19. In the present study, our results revealed that miR‐222 promoted the proliferation of bladder cancer cells *in vitro*. Moreover, miR‐222 attenuated CDDP‐induced cell death in bladder cancer. Our findings revealed that PPP2R2A is a direct target of miR‐222 in bladder cancer cells. The PPP2R2A/Akt/mTOR axis was required for miR‐222‐induced proliferation and CDDP resistance in bladder cancer cells.

The role of miR‐222 in cancer cells remains controversial. miR‐222 has been considered to be either an onco‐miR or a tumour suppressor miR 8. In bladder cancer, high levels of miR‐222 are associated with a poor prognosis, indicating that miR‐222 may be an onco‐miR in bladder cancer 5. In our study, we also observed that miR‐222 promoted proliferation in bladder cancer cells *in vitro*, supporting the hypothesis that miR‐222 played an onco‐miR role in bladder cancer cells.

Overexpression of miR‐222 *in vitro* down‐regulated the expression of PPP2R2A in bladder cancer cells. PPP2R2A belongs to the PP2A regulatory subunit B family, which is one of the variable regulatory subunits of PP2A 20. In cancer cells, decreased PP2A activity induces the activation of various kinases related to proliferation, and thus promotes tumour progression 10, 11, 12, 13, 14, 15. PP2A regulates many intracellular processes, including cellular signalling, the cell cycle, metabolism, apoptosis and protein synthesis 21, 22, 23. Akt is one of the substrates of PP2A, and miR‐222‐induced down‐regulation of PPP2R2A is associated with the activation of the Akt pathway 4, 24, 25, 26, which in turn can activate the mTOR pathway. The activation of the PI3K/Akt/mTOR pathway is associated with tumour growth 27, 28, whereas the inactivation of Akt and mTOR suppresses tumour growth 29. In hepatocellular carcinoma cells, miR‐222‐induced down‐regulation of PPP2R2A is associated with Akt activation 4. Therefore, in the present study, we considered PPP2R2A to be as a direct target of miR‐222. We observed a significant activation of Akt/mTOR in miR‐222‐overexpressing bladder cancer cells. In contrast, blocking the phosphorylation of Akt or mTOR prevented miR‐222‐induced proliferation. Therefore, our data demonstrated that the PPP2R2A/Akt/mTOR axis played a regulatory role in the miR‐222‐induced proliferation of bladder cancer. Some studies have reported that miR‐222 targets other tumour suppressors, such as p27 30. Although we did not investigate these alterations, these tumour suppressors may also contribute to miR‐222‐induced proliferation and CDDP resistance.

Overexpression of miR‐222 has been reported to be associated with chemotherapy 8. In breast cancer cells, miR‐222 down‐regulates ERα, whereas knock‐down of miR‐222 sensitizes MDA‐MB‐468 cells to tamoxifen treatment 31. Knock‐down of miR‐222 restores sensitive sensitivity to TRAIL in TRAIL‐resistant NSCLC cells 32. In our study, we observed that overexpression of miR‐222 in bladder cancer cells antagonized CDDP‐induced cell death, whereas its knock‐down enhanced the antitumour effects of CDDP. Inhibition of PI3K/Akt activation enhanced sensitivity to CDDP 33. Interestingly, our data showed that blockade of Akt or mTOR activation restored sensitivity to CDDP upon miR‐222 overexpression. Therefore, these results indicated that the PPP2R2A/Akt/mTOR axis was required for miR‐222‐mediated CDDP resistance in bladder cancer.

CDDP is a known cytotoxic drug that can induce antitumorigenic autophagy and thus result in type II programmed cell death in cancer cells 16, 34, 35. In the present study, we observed that overexpression of miR‐222 in bladder cancer cells suppresses autophagy; this suppression might be mediated by the activation of the Akt/mTOR signalling pathway. Although the exact role of miR‐222‐mediated autophagy inhibition in CDDP‐induced cell death remains unclear, our study provides evidence that miR‐222 is a novel anti‐autophagy microRNA in bladder cancer cells. We also postulate that the inhibition of autophagy may contribute (at least partially) to miR‐222‐induced CDDP resistance in bladder cancer cells.

Together, our results indicate that miR‐222 promotes the proliferation of bladder cancer cells. Importantly, our study highlighted for the first time a novel miR‐222/PPP2R2A/Akt/mTOR axis that regulates CDDP sensitivity in bladder cancer. Therefore, miR‐222 may be a novel potential target for the treatment of bladder cancer and chemotherapy drug resistance.

## Conflicts of interest

The authors declare no conflict of interest.
